# Thrombosis after liver transplantation for hepatocellular carcinoma

**DOI:** 10.1371/journal.pone.0186699

**Published:** 2017-10-26

**Authors:** Ida Martinelli, Francesca R. Ponziani, Alberto Maino, Sherrie Bhoori, Maria Abbattista, Umberto Maggi, Tullia M. De Feo, Paolo Bucciarelli, Andrea Artoni, Elena Longhi, Marta Serafini, Giorgio Rossi, Vincenzo Mazzaferro

**Affiliations:** 1 A. Bianchi Bonomi Hemophilia and Thrombosis Center, Fondazione IRCCS Ca' Granda—Ospedale Maggiore Policlinico, Milan, Italy; 2 Liver Surgery, Transplantation and Gastroenterology, University of Milan and Istituto Nazionale Tumori Fondazione IRCCS, Milan, Italy; 3 Hepatobiliary and Liver Transplant Unit, Fondazione IRCCS Ca' Granda—Ospedale Maggiore Policlinico and University of Milan, Milan, Italy; 4 North Italy Transplant program (NITp), Organ and Tissue Transplant Immunology, Fondazione IRCCS Ca' Granda—Ospedale Maggiore Policlinico, Milan, Italy; Universita degli Studi di Pisa, ITALY

## Abstract

The influence of thrombosis on the prognosis of patients with hepatocellular carcinoma (HCC) after liver transplantation (LT) and the role of the commonest inherited thrombophilia abnormalities factor V Leiden and prothrombin G20210A in the development of thrombosis are unknown. We investigated a cohort of patients who underwent LT for HCC with the aim to estimate the incidence rate (IR) of thrombosis, its influence on mortality and re-transplantation rates and, in the frame of a nested case-control study, the role of thrombophilia in donors and recipients for the development of thrombosis. Four-hundred and thirty patients underwent LT and were followed for a median of 7.2 years. Twenty-six recipients (6%) developed thrombosis (IR 1.06 [95%CI: 0.71–1.53] per 100 pts-yr). Mortality rate after LT was 3.95 (95%CI: 3.22–4.79) per 100 pts-yr and was not influenced by thrombosis. Re-transplantation was planned for 33 patients and was more common in patients with thrombosis than in those without (HR 2.50 [95%CI: 0.87–7.17]). The risk of thrombosis was 4 times higher in recipients with thrombophilia than in those without (OR 4.23 [95%CI: 0.99–18.04]) and 6 times higher when the analysis was restricted to venous thrombosis (OR 6.26 [95%CI: 1.19–32.85]). The presence of inherited thrombophilia in the donors did not increase the risk of thrombosis of the recipient. In conclusion, thrombosis is a complication of 6% of patients transplanted for HCC and increases the risk of re-transplantation but not of mortality. The risk of thrombosis, particularly venous, is increased in the presence of thrombophilia abnormalities in the recipients.

## Introduction

Hepatocellular carcinoma (HCC) is the fifth most common tumour worldwide and the second most common cause of cancer-related death [1]. Despite the introduction of HBV vaccination and new direct-acting antiviral drugs against HCV hepatitis, the incidence of HCC is increasing sharply [2] and is supposed to rise continuously [3]. The prognosis of HCC has improved over the past two decades, especially in developed countries, where 30–40% of cases are now diagnosed at early stages when curative treatments can be proposed [4, 5]. An 80% 5-yr survival can be achieved by transplanting selective patients within the Milan Criteria [6–8].

Thrombosis is a life-threatening complication of LT, and it can have a deep impact on both graft and patients’ survival [9]. Thrombosis often occurs in the immediate post-operative period involving mainly the hepatic artery, but also the portal vein, the inferior vena cava and the hepatic vein system may be affected as well [9–13]. While hepatic artery thrombosis is mainly attributable to surgical procedures [9, 14], portal vein thrombosis can also be a consequence of a small portal vein size (<5mm) and of a previous splenectomy [15]. To date, data on thrombosis after LT are scarce, with an incidence ranging from 1% to 15% in various case series, mainly describing pediatric patients [9, 15, 16].

Like most cancers, HCC is associated with activation of the haemostatic system leading to an increased risk of venous and arterial thrombosis [17]. Also the inherited thrombophilia abnormalities, factor V Leiden and the G20210A mutation in the prothrombin gene, common in individuals of Caucasian origin, are associated with a 3 to 8 fold increase in the risk of thrombosis [18, 19]. Their prevalence in the general population is 3–8% for factor V Leiden and 2% for the prothrombin G20210A mutation [18]. The two mutations have already been associated with a 2 to 4-fold increased risk of venous thrombosis in patients suffering from cancer of all histotypes [20] and they have also been implicated in the development of thrombosis in the post-transplant period [21–23]. A recent review of the literature provide insights on the risk of thrombosis in patients undergoing LT [24], but studies including patients with HCC were intentionally excluded. Limited and weak data is available in patients undergoing LT for HCC. No data is available in patients undergoing LT for HCC.

Aims of this study were to evaluate the incidence rate of thrombosis in a cohort of patients with HCC before and after LT, its impact on patients’ survival and rate of re-transplantation. In addition, the role of factor V Leiden and prothrombin G20210A mutation on the development of thrombosis was investigated.

## Patients and methods

### Patients

Patients with HCC enlisted for LT between 2001 and 2013 in two liver transplant Units both in Milan, Istituto Nazionale Tumori and Ospedale Maggiore Policlinico, and both referring to the North Italy Transplant Program (NITp) were included in this retrospective study cohort. The NITp Reference Center, other than being in charge of organ allocation, is responsible for performing pre-transplant immunological testing [25]. Data were collected from medical records and included demography, date of entry in (or entries for re-transplanted patients) and date and reason for exiting the waiting list, date of LT, tumour-related, liver disease-related, surgery-related variables, date and causes of death. Such information were obtained by the medical staff at the two liver transplant Units and matched and merged with those collected by the NITp.

Patients’ follow-up was split into two periods, before and after LT, as the main pre-operative risk for thrombosis is the presence of HCC while the post-operative risk is represented by the surgical intervention (for early thrombosis) and the graft’s function. The first period included time from the enlisting to the date of LT (or drop-out); the second period included time from the enlisting to the date of death, thrombosis or till July 31^st^, 2015 (administrative censoring). Patients with worsening clinical conditions were considered for re-transplantation and were eventually re-listed. During the entire follow-up, the occurrence of arterial or venous thrombosis at any site were recorded. Antiplatelet therapy was routinely prescribed after LT to all patients. To investigate the role of factor V Leiden and prothrombin G20210A mutation on the risk of thrombosis after LT, a nested case-control study was performed, with patients who developed thrombosis as cases. For each case, three patients who did not develop thrombosis after LT were randomly selected as controls. Thrombosis was defined “early” when it occurred within 1 month from LT and “late” when occurred thereafter [26]. Additional information on recurrence of HCC and complexity of LT were retrieved from medical records. “Complex LT” was defined when one or more of the following conditions occurred: 1) multiple vascular anastomosis (and use of jumping grafts), aberrant donor or recipient arterial anatomy and complex back-table vascular reconstructions; 2) length of surgery (exceeding the 75^th^ percentile of the transplantation time the whole cohort); 3) extensive use of blood products (exceeding the 75^th^ percentile of blood products transfused to the whole cohort); 4) recipients’ MELD (Model for End-Stage Liver Disease) score >29; 5) presence of portal vein thrombosis of the main trunk before transplantation.

The Ethic Committee of Milano Area B and the Ethic Committee of Istituto Nazionale dei Tumori approved the study and waived the need for informed consent.

### Laboratory tests

DNA samples from recipients and donors stored at NITp’s biobank at the Ospedale Maggiore Policlinico were obtained for patients belonging to the nested case-control study. DNA was isolated from frozen peripheral blood collected at the time of the enlisting for recipients and of organ donation for donors. DNA purification was performed with Qiagen EZ1 Advance XL automated instrument using EZ1 DSP DNA Blood Kit, version 3 (QIAGEN GmbH, QIAGEN Strasse 1, 40724 Hilden, Germany), obtaining a DNA concentration range from 25 to 100 ng/μl. DNA analyses for the 1691 G>A substitution in FV (FV Leiden) and for the 20210 G>A substitution of the prothrombin genes were performed according to previously described methods at the laboratory of the Thrombosis Center at the Ospedale Maggiore Policlinico [18].

### Statistical analysis

In the cohort study, median follow-up after LT was calculated using the estimates of the censoring distribution as previously described [27]. The incidence rates (IR) of arterial and venous thrombosis, death and the necessity of re-transplantation were calculated with their 95% confidence intervals (CI) according to Poisson distribution for the periods before and after LT, and expressed as events per 100 patients-year (pts-yr). Recipients who developed thrombosis were compared to those who did not for overall mortality and the need of re-transplantation in terms of incidence rate ratios (IRR). Hazard ratios (HR) and their 95% CI, obtained by a Cox proportional hazard regression model and adjusted for sex, age and year of LT, were also calculated. Kaplan-Meier curves were used to plot the cumulative incidence of thrombosis after LT.

For the nested case-control study, cases and controls were matched for the year of LT, i.e., controls were chosen randomly among patients transplanted in the same calendar year as the corresponding cases. Assuming an 8% overall prevalence of factor V Leiden and prothrombin G20210A mutation in the control group [18, 19] and a 4-times higher risk of venous thrombosis in carriers than non-carriers [20], to obtain a power of 80% with an alpha error of 5%, 36 cases and 108 controls would be needed. With an a priori cumulative incidence of thrombosis after LT of 15% at 5 years, the inclusion of 400 transplanted patients in the cohort would be sufficient to observe the required number of cases. Cases and controls were compared for the presence of the mutations both in donors and recipients in terms of odds ratio (OR) and 95% CI. OR were obtained by a conditional logistic regression model, and adjusted for age at LT, sex and BMI. Subgroups analyses were performed for venous and arterial thrombosis and for early and late thrombosis. All analyses were performed with the statistical software SPSS (release 23.0, IBM SPSS Statistics for Windows, IBM Corp., Armonk, NY, USA).

## Results

**Fig 1:**
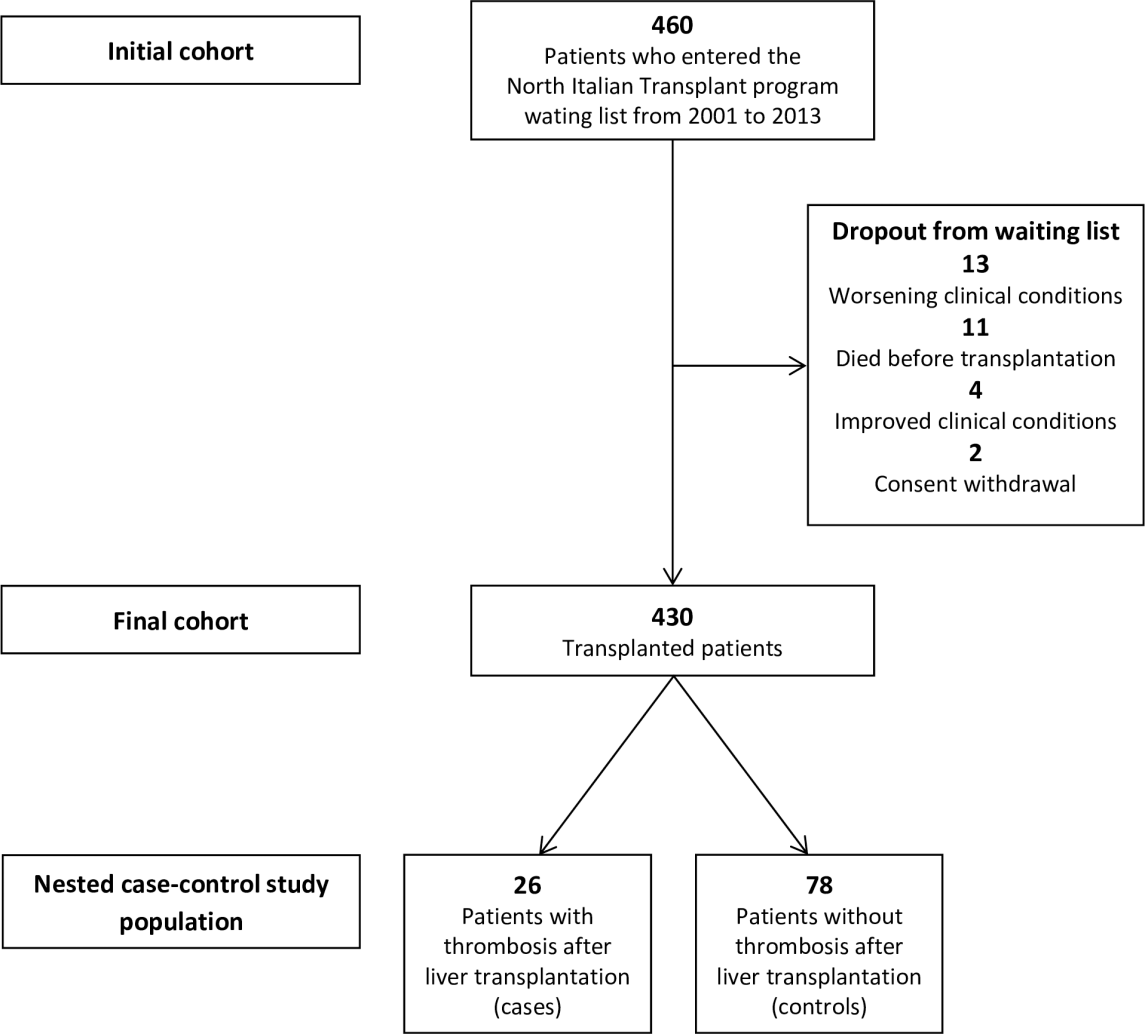
Flow-chart of the study population of patients with hepatocellular carcinoma.

The whole cohort was formed by 460 patients (Fig 1). Thirty patients (6.5%) exited the list because of worsening of clinical conditions (n = 13), death (n = 11), clinical improvement (n = 4) or consent withdrawal (n = 2).

Hence, 430 patients underwent LT (305 at Istituto Nazionale Tumori and 125 at Ospedale Maggiore Policlinico). In the first follow-up period, the median time from the inclusion in the list to LT was 2.6 months (IQR 1.3–4.9) and no thrombosis occurred in this time frame. Demographic and clinical characteristics at the time of LT are shown in Table 1.

**Table 1:** Characteristics of patients who underwent liver transplantation.

Characteristics	Transplanted patients
**N°of patients**	**430**
Men/women	379/51
Age at liver transplantation, mean (SD)	55.4 (6.4)
Time in waiting list (months), mean (SD)	4.2 (5.9)
Body mass index (kg/m^2^), mean (SD)	25.4 (3.7)
**Blood group, n (%)**	
O	178 (41.4)
Non-O	252 (58.6)
**Cause of cirrhosis, n (%)**	
HBV infection	78 (18.1)
HBV + other cause	35 (8.1)
HCV infection	137 (31.9)
HCV + other cause	86 (20)
Coinfections (HBV, HCV)	24 (5.6)
Alcohol	38 (8.8)
Cryptogenetic	22 (5.1)
Other causes	10 (2.3)
**MELD score at liver transplantation, median (range)**	9 (8–11.8)
**Child-Pugh score at liver transplantation, n (%)**	
A	232 (53.9)
B	79 (18.4)
C	15 (3.5)
**Tumor characteristics**	
**Nodules at liver transplantation, n (%)**	
≤3	346 (80.5)
>3	65 (15.8)
**Size of largest tumor mass at liver transplantation (cm), median (min, max)**	2.5 (0–9)
**Liver characteristics, n (%)**	
Full size graft	407 (94.7)
Split/Living donor	23 (5.3)

SD, standard deviation; HBV, hepatitis B virus; HCV, hepatitis C virus; HDV, hepatitis D virus; MELD, Model for End-Stage Liver Disease.

In the second period of observation, follow-up was complete for all patients. The overall median follow-up time after LT was 7.17 years (95%CI 6.78–7.56) for a total of 2449 pts-yr. Twenty-six patients (6%) developed thrombosis (19 venous and 7 arterial) for an IR of 1.06 (95%CI 0.71–1.53) per 100 pts-yr (0.76, 95%CI 0.46–1.19 for venous thrombosis and 0.27, 95%CI 0.11–0.57 for arterial thrombosis). Thrombosis occurred after a median time of 19 days (IQR 12–731) from LT, with a higher incidence of early thrombosis (14/26, IR 9.5, 95%CI 8.0–11.2 per 100 pts-yr) than late thrombosis (12/26, IR 0.07, 95%CI 0.04–0.13 per 100 pts-yr).

**Fig 2:**
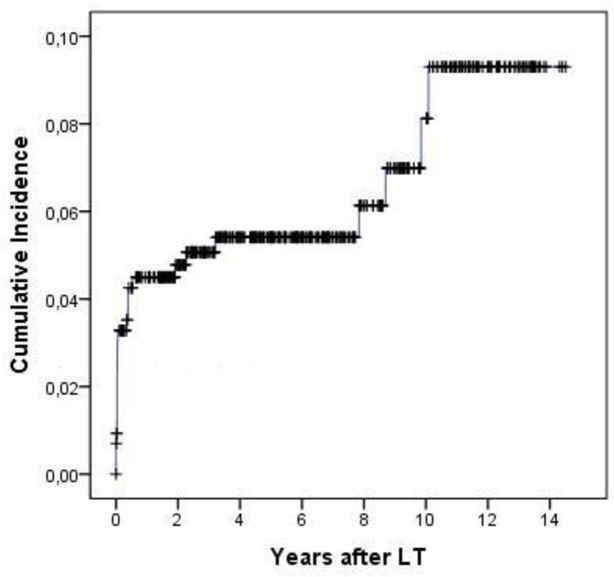
Kaplan Meier curve for cumulative incidence of thrombosis after liver transplantation.

As shown in Fig 2, the cumulative incidence of thrombosis at 1 month, 1 year and 5 years was 3%, 4% and 5%, respectively. After LT 101 patients died, for an overall mortality rate of 3.95 (95%CI 3.22–4.79) per 100 pts-yr and a survival rate at 5 years of 78%. Mortality was similar in patients with and without thrombosis (adj. HR 0.92, 95%CI 0.41–2.11) (Table 2).

**Table 2:** Incidence rates of mortality and need of re-transplantation in patients with or without thrombosis.

	N.	pts-yr	IR per 100 pts-yr (95% CI)	IRR	Adj.HR* (95% CI)
**Mortality after liver transplantation**					
**All recipients**	101	2557.4	3.95 (3.22–4.79)		
**without thrombosis**	95	2402.3	3.95 (3.19–4.83)	Ref.	Ref.
**with thrombosis**	6	155.1	3.87 (1.42–8.42)	0.98 (0.43–2.23)	0.92 (0.41–2.11)
**Planned re-transplantation**					
**All recipients**	33	2441.0	1.35 (0.93–1.89)		
**without thrombosis**	28	2312.9	1.21 (0.80–1.75)	Ref.	Ref.
**with thrombosis**	5	128.0	3.91(1.27–9.11)	3.23 (1.25–8.35)	2.50 (0.87–7.17)

pts-yr, patients year; IR, incidence rate; CI, confidence intervals; IRR, incidence rate ratio; Ref, reference.

*Adjusted for sex, age and year at liver transplantation.

Re-transplantation was considered as a viable therapeutic option in 33 patients, for an IR of 1.35 (95%CI 0.93–1.89) per 100 pts-yr. Thrombosis was the reason for re-transplantation in five patients (15%); the hepatic artery was the site of thrombosis in two patients, the portal vein in three. The incidence of re-transplantation was 3-times higher in patients with thrombosis than in those without (IRR 3.23, 95% CI 1.25–8.35) (Table 2).

The nested case-control study included 26 patients with thrombosis and 78 without. Demographic and baseline clinical characteristics of cases and controls are shown in Table 3. Sex, age and clinical characteristics did not substantially differ between groups. HCC recurred in 5 cases (19%) and 23 controls (29%). In only one case recurrence preceded thrombosis. Complex LT was recorded in 7 cases (27%) and 29 controls (37%). Among the former, 5 had an early venous thrombosis, one a late arterial and one a late venous thrombotic event. Thrombosis of the hepatic artery was mainly distributed in the early period after LT (71% of the events), whereas venous thrombosis was similarly distributed in the early and late periods (47% e 53% of the events). Four patients (4/26, 15%) developed proximal deep vein thrombosis of the lower or upper limbs or pulmonary embolism, two in the first month after LT and two later. A detailed description of each thrombotic event is shown in supplemental Table in S1 Table.

**Table 3:** Characteristics of patients included in the nested case-control study.

	Cases n = 26	Controls n = 78
Male, n (%)	22 (85)	62 (79)
Age at liver transplantation, median (IQR)	57 (52–61)	56 (50–59)
Body mass index, mean (sd)	26.2 (3.3)	25.5 (3.6)
**Tumor-related variables**		
N of nodules, median (IQR)	2 (1–3)	2 (1–3)
Tumor size, median (IQR)	2.8 (1.8–5.0)	2.9 (2.0–4.0)
Milan IN, n (%)	19 (73)	58 (74)
Microvascular invasion, n (%)	11 (42)	23 (29.5)
Grading, median (IQR)	2 (1–3)	2 (1–3)
Satellite lesions, n (%)	2 (7.7)	5 (6.4)
AFP>400 at liver transplantation, n (%)	0	2 (2.6)
Downstaging procedures, n (%)	7 (27)	7(9)
**Liver disease-related variables**		
MELD score, median (IQR)	8.6 (7.4–11.5)	8 (7.5–11)
Child Pugh score, n (%)		
A	14 (53.8)	46 (58.9)
B	6 (23.1)	15 (19.2)
C	0	7 (8.9)
**Cause of cirrhosis, n (%)**		
HBV infection	3 (11.5)	17 (21.8)
HBV + other cause	3 (11.5)	4 (5.1)
HCV infection	10 (38.5)	32 (41)
HCV + other cause	2 (7.7)	11 (14.1)
Coinfections (HBV, HCV)	3 (11.5)	3 (3.8)
Alcohol	4 (15.4)	6 (7.7)
Cryptogenetic	1 (3.8)	2 (2.6)
Other causes	0	3 (3.8)
**Surgery-related variables**		
Transfused patients, n° (%)	24 (92)	70 (89.7)
- Red blood cells	17	47
- Platelets	3	5
- Fresh frozen plasma	22	68
Surgery duration >10hrs, n (%)	2 (7.7)	8 (10.3)
Cold ischemic time, median (IQR)	435 (387.5–512.5)	410 (370–480)
Complex liver transplantation, n (%)	7 (27)	29 (37)
**Matching-related variables**		
ABO identical, n (%)	25 (96.2)	75 (96.2)
Whole liver/split, n	24/2	75/3
Donor age, median (IQR)	64 (49–76)	62 (49–71)
Post–operative acute rejection req. steroids, n (%)	0	2 (2.6)

SD, standard deviation; IQR, interquartile range; AFP, alpha-fetoprotein; HBV, hepatitis B virus; HCV, hepatitis C virus; HDV, hepatitis D virus; MELD, Model for End-Stage Liver Disease.

Among recipients, factor V Leiden or prothrombin G20210A mutation were found in 5 cases (19.2%) and 4 controls (5.1%) and carriers had a 4-fold increased risk of thrombosis, that increased to 6.5-fold when only venous thrombosis was considered (Table 4).

**Table 4:** Association between thrombophilia (heterozygous factor V Leiden or prothrombin G20210A mutation) in the recipients and the risk of thrombosis after liver transplantation.

	Cases n = 26	Controls n = 78	Crude OR (95% CI)	Adj.OR* (95% CI)
**All thrombosis**				
**Thrombophilia negative, n (%)**	21 (80.8)	74 (94.9)	Ref.	Ref.
**Thrombophilia positive, n (%)**	5 (19.2)	4 (5.1)	4.23 (0.99–18.04)	4.25 (0.95–18.96)
**Venous thrombosis**				
**Thrombophilia negative, n (%)**	14 (74)	74 (95)	Ref.	Ref.
**Thrombophilia positive, n (%)**	5 (26)	4 (5)	6.26 (1.19–32.85)	7.46 (1.26–43.99)

OR,odds ratio; CI, confidence intervals; Ref, reference.

*Adjusted for age at liver transplantation, sex and body mass index.

The association was present both for early (OR 6.0, 95%CI 0.54–66.17) and late (OR 3.41, 95%CI 0.55–21.13) thrombosis. Among donors prothrombin G20120A mutation was found in one case and two controls for an OR of 1.64 (95%CI 0.14–19.03), while none had factor V Leiden. One recipient with factor V Leiden whose donor had prothrombin mutation developed an extrahepatic portal vein thrombosis 10 years after LT.

## Discussion

In this large cohort of patients who underwent LT for HCC the overall incidence rate of thrombosis was 1.06 per 100 pts-yr, with more than half of the events occurring in the first month after LT regardless of the complexity of surgery. Thrombosis was associated with a 3-fold increased risk of re-transplantation, but it did not influence the overall mortality. Two-thirds of the thrombotic events after LT were venous, and thrombophilia abnormalities factor V Leiden or prothrombin G20210A mutation conferred an increased risk of thrombosis. Recipients carrying mutations had a 6.5-fold increased risk of venous thrombosis compared to non-carriers.

Differently from previous observations [9, 11, 12] we found a higher incidence of venous rather than arterial thrombosis. This might reflect the different baseline risk profile of patients who undergo LT for malignancy rather than for other non-tumoral etiologies and may also be attributed to the antiplatelet therapy that is routinely given in the two liver transplant Units after surgery. Another difference between our and other cohort studies performed in children and adults [9, 23, 26] is the finding that thrombosis was associated with a 3-fold increased risk of re-transplantation but it did not influence overall mortality. Our cohort is made of adult transplanted HCC patients and this peculiarity is probably responsible for such differences, thus making comparisons with previous data inappropriate. Concerning thrombophilia, two studies failed to find an association between the two mutations and thrombosis after LT for various indications [21, 23]. Both studies however, included pediatric patients and observed mainly arterial thrombosis. One study, similarly to ours, investigated the presence of the mutations also in the grafts, failing to find an association with thrombosis [21]. Hence, testing factor V Leiden and prothrombin G20210A mutation in the donors seems worthless to prevent thrombosis in the recipients. There could be different reasons to explain why thrombophilia abnormalities of the recipients may be associated with an increased risk of thrombosis, being factor V and prothrombin synthesized by the liver. Factor V Leiden exerts its pro-thrombotic potential causing resistance of coagulation factor V to the inactivation operated by its natural anticoagulant activated protein C [18] and the prothrombin G20210A mutation increases, through mechanisms that are not fully elucidated, plasma levels of prothrombin [18]. In the setting of transplantation, we can hypothesise that thrombophilia abnormalities, particularly factor V Leiden, influence the risk of thrombosis through three different mechanisms. First, a transplanted liver from a thrombophilic donor can chronically enhance fibrin deposition, therefore predisposing to thrombosis [28]. Second, fibrin deposition of the recipient’s liver with HCC may last after LT. Third, being 10 to 20% of circulating factor V stored in platelets and factor V mRNA contained in megakariocytes, it is possible that part of intraplatelet factor V of the recipient carrier of factor V Leiden favours a prothrombotic phenotype [29]. Our results support the latter two hypothesis.

To our knowledge, this is the first study of patients with HCC followed after LT and in whom thrombophilia abnormalities were searched both in recipients and donor grafts. Among the strengths of the study, there is a single cohort of patients generated from two liver transplant Units that share the same attitude on patient/tumor selection, the same surgical skills and the same reference center for organ allocation and immunological testing. The retrospective design offers the unique advantage to collect data from a large cohort of patients with a long follow-up, goal hardly achievable with a prospective design; on the other hand, missing data or errors are obviously more probable in retrospective than in prospective studies. However, these limitations are minimized in this study, as most of the data were accurately collected and all patients strictly followed-up throughout lifetime, according to the protocol carried out in most transplant centers worldwide. In addition, even if a large number of patients formed the initial cohort, the predicted sample of 36 patients with thrombosis was not reached, because the incidence of thrombosis was lower than expected. However, the observed association between venous thrombosis and thrombophilia appeared stronger than predicted, leading to an acceptable statistical power despite the relatively low number of patients with thrombosis.

In conclusion, 6% of patients with HCC develop thrombosis after LT, with an incidence rate of 1.06 per 100 pts-yr. Thrombosis occurs mainly in the first month after transplantation and affects graft survival but not overall mortality. The common thrombophilia abnormalities factor V Leiden or prothrombin G20210A mutation in the recipients may increase the risk of venous thrombosis. Whether recipients should be tested for thrombophilia and those carrying thrombophilia abnormalities benefit from a more aggressive antithrombotic prophylaxis needs to be addressed in further studies.

## Supporting information

S1 Table Detailed description of thrombosis and thrombophilia. (DOCX)

S1 Data LT in HCC data file. (XLSX)

## Abbreviations

CI: confidence interval
FV: factor V Leiden
HCC: hepatocellular carcinoma
HR: hazard ratio
IQR: interquartile range
IR: Incidence rate
IRR: incidence rate ratio
LT: liver transplantation
MELD: Model for End-Stage Liver Disease
OR: odds ratio
pts-yr: patients-year
SD: standard deviation
