# Supplementary material for: Thrombosis after liver transplantation for hepatocellular carcinoma
Source: PLoS One. 2017 Oct 26;12(10):e0186699. doi: 10.1371/journal.pone.0186699 (PMC5658078; doi:10.1371/journal.pone.0186699)
Supplement: S1 Table — (DOCX) [file pone.0186699.s001.docx]

**Table S1.** Detailed description of thrombosis and thrombophilia.

| # | **Days after LT** | **Thrombophilia** | | | | **Type** | **Site** | **Complex LT** |
| --- | --- | --- | --- | --- | --- | --- | --- | --- |
|  |  | **Recipient** | | **Donor** | |  |  |  |
|  |  | **FVL** | **G20210A** | **FVL** | **G20210A** |  |  |  |
| **1** | 0 |  |  |  |  | A | hepatic artery |  |
| **2** | 2 |  |  |  |  | V | extrahepatic portal vein |  |
| **3** | 2 |  |  |  |  | V | right hepatic vein + inferior vena cava | ✓ |
| **4** | 5 |  |  |  |  | V | right hepatic vein + inferior vena cava | ✓ |
| **5** | 11 |  |  |  |  | A | hepatic artery |  |
| **6** | 12 |  | ✓ |  |  | V | inferior vena cava | ✓ |
| **7** | 12 |  |  |  |  | A | hepatic artery |  |
| **8** | 12 |  |  |  |  | A | hepatic artery |  |
| **9** | 12 |  | ✓ |  |  | V | left popliteal vein + pulmonary embolism |  |
| **10** | 12 |  |  |  |  | V | right hepatic vein | ✓ |
| **11** | 14 |  |  |  |  | A | hepatic artery |  |
| **12** | 15 |  |  |  |  | V | extrahepatic portal vein | ✓ |
| **13** | 16 |  |  |  |  | V | extrahepatic portal vein |  |
| **14** | 22 |  |  |  |  | V | pulmonary embolism |  |
| **15** | 126 | ✓ |  |  |  | V | right hepatic vein + inferior vena cava |  |
| **16** | 139 |  |  |  |  | V | right and right posterior portal vein |  |
| **17** | 141 |  |  |  |  | A | hepatic artery | ✓ |
| **18** | 141 |  |  |  |  | V | left hepatic vein |  |
| **19** | 191 |  | ✓ |  |  | V | extrahepatic portal vein |  |
| **20** | 698 |  |  |  |  | V | right hepatic vein |  |
| **21** | 830 |  |  |  |  | A | hepatic artery |  |
| **22** | 1169 |  |  |  |  | V | right and left portal vein |  |
| **23** | 2864 |  |  |  |  | V | right popliteal vein + pulmonary embolism |  |
| **24** | 3178 |  |  |  |  | V | hepatic veins | ✓ |
| **25** | 3596 | ✓ |  |  | ✓ | V | extrahepatic portal vein |  |
| **26** | 3683 |  |  |  |  | V | right subclavian vein |  |

LT=liver transplantation; FVL=factor V Leiden; G20210A=prothrombin mutation; A=arterial thrombosis; V=venous thrombosis
